# Costs and consequences of large-scale vector control for malaria

**DOI:** 10.1186/1475-2875-7-258

**Published:** 2008-12-17

**Authors:** Joshua O Yukich, Christian Lengeler, Fabrizio Tediosi, Nick Brown, Jo-Ann Mulligan, Des Chavasse, Warren Stevens, John Justino, Lesong Conteh, Rajendra Maharaj, Marcy Erskine, Dirk H Mueller, Virginia Wiseman, Tewolde Ghebremeskel, Mehari Zerom, Catherine Goodman, David McGuire, Juan Manuel Urrutia, Fana Sakho, Kara Hanson, Brian Sharp

**Affiliations:** 1 Department of Public Health and Epidemiology, Swiss Tropical Institute, P.O. Box, 4002 Basel, Switzerland; 2 Centre for Research on Health and Social Care Management, Università Bocconi, Milan, Italy; 3 ITN Cell, National Malaria Control Programme, Ministry of Health, Dar Es Salaam, Tanzania; 4 Health Economics and Financing Programme, London School of Hygiene and Tropical Medicine, Keppel Street, London, WC1E 7HT, UK; 5 Population Services International, Nairobi, Kenya; 6 Medical Research Council Laboratories, Banjul, Gambia; 7 Population Services International, Blantyre, Malawi; 8 Malaria Lead Programme, Medical Research Council of South Africa, Durban, KwaZulu-Natal, South Africa; 9 Canadian Red Cross, Ottawa, Ontario, Canada; 10 National Malaria Control Programme, Ministry of Health, P.O. Box 212, Asmara, Eritrea; 11 KEMRI/Wellcome Trust Programme, PO Box 43640, Nairobi, Kenya; 12 Abt Associates Inc., Bethesda, MD, USA; 13 NetMark Partnership, Academy for Educational Development, Johannesburg, South Africa; 14 NetMark Partnership, Academy for Educational Development, Dakar, Senegal

## Abstract

**Background:**

Five large insecticide-treated net (ITN) programmes and two indoor residual spraying (IRS) programmes were compared using a standardized costing methodology.

**Methods:**

Costs were measured locally or derived from existing studies and focused on the provider perspective, but included the direct costs of net purchases by users, and are reported in 2005 USD. Effectiveness was estimated by combining programme outputs with standard impact indicators.

**Findings:**

Conventional ITNs: The cost per treated net-year of protection ranged from USD 1.21 in Eritrea to USD 6.05 in Senegal. The cost per child death averted ranged from USD 438 to USD 2,199 when targeting to children was successful.

Long-lasting insecticidal nets (LLIN) of five years duration: The cost per treated-net year of protection ranged from USD 1.38 in Eritrea to USD 1.90 in Togo. The cost per child death averted ranged from USD 502 to USD 692.

IRS: The costs per person-year of protection for all ages were USD 3.27 in KwaZulu Natal and USD 3.90 in Mozambique. If only children under five years of age were included in the denominator the cost per person-year of protection was higher: USD 23.96 and USD 21.63. As a result, the cost per child death averted was higher than for ITNs: USD 3,933–4,357.

**Conclusion:**

Both ITNs and IRS are highly cost-effective vector control strategies. Integrated ITN free distribution campaigns appeared to be the most efficient way to rapidly increase ITN coverage. Other approaches were as or more cost-effective, and appeared better suited to "keep-up" coverage levels. ITNs are more cost-effective than IRS for highly endemic settings, especially if high ITN coverage can be achieved with some demographic targeting.

## Background

Prevention of malaria in highly endemic countries relies largely on vector control through one of two main methods: insecticide treated (mosquito) nets (ITNs) and indoor residual (house) spraying (IRS). Both methods are known to be highly effective and current evidence suggests they are very similar in their impact [1]. Given the increasing availability of resources for malaria control, the Roll Back Malaria Partnership (RBM) has set the ambitious target for 2010 of 80% protection of high-risk groups by a "locally appropriate" vector control measure [2]. While few countries were near this objective in 2007, substantial progress has been made. Inevitably, this has been accompanied by vigorous debate as to the best way forward with regard to the different implementation models for ITNs, as well as the relative merits of ITNs versus IRS [3-8]. While the implementation of IRS is typically through vertical programmes, available options for ITN implementation are more diverse.

Besides feasibility, sustainability and health impact, cost is obviously an important factor in the choice between different strategic options. Unfortunately, little is known on comparative costs and operational requirements for the delivery of ITNs and IRS. Direct comparisons in single settings in sub-Saharan Africa (SSA) have shown conflicting results on cost per person protected using either control method [9-11]. A comprehensive modelling study has been conducted covering ITNs and IRS [12] and the authors found overlapping cost-effectiveness ranges for the two interventions. For ITNs, four studies have examined large-scale programmes using field data in The Gambia, Malawi, Tanzania and Togo [13-16]. However, each of these studies focused on only one strategy and methodological differences make direct comparisons difficult [17]. With the scale up of ITN activities in several SSA countries it has become possible to collect and analyse such information for a range of settings. This was undertaken for ITNs in five sub-Saharan countries, and for IRS in two countries. The present work allows, for the first time, a direct cost-effectiveness comparison between different ITN strategies and IRS implementation on a large scale. Hence, this work provides a solid evidence base for a discussion of malaria vector control strategies in the most highly malarious areas of the world.

## Methods

### Programme selection

The ITN programmes were deliberately chosen to represent the major existing distribution strategies operating at large or fully national scale in sub-Saharan Africa. At the beginning of this research, few large-scale ITN programmes utilizing similar distribution systems existed, thus the choice of a representative country for each strategy was limited. The main exception was subsidized commercial distribution, which was implemented in several countries. Senegal was chosen for this category mainly to increase representation of West African countries. The strategies were defined using terminology derived from Webster *et al *[18]: (1) free ITN delivery through public sector health services and at the community level – Eritrea [19,20]; (2) free public sector ITN delivery through integrated vaccination campaigns – Togo [21]; (3) highly subsidized mixed public-private sector ITN delivery through routine services – Malawi [22]; (4) partially subsidized private retail sector promotion – Senegal [23]; (5) partially subsidized private retail sector promotion with a partially subsidized, mixed public-private, routine services voucher scheme – Tanzania [24]. Table 1 presents programme details with regard to size, time frame, total economic costs and the latest available coverage figures.

**Table 1:** Main characteristics of the ITN and IRS programmes that were reviewed.

	**Population covered** **(millions)**	**Period**	**Total number of nets**	**Total number of re-treatments**	**Total economic cost** **(Mio USD)**	**Net coverage in children under five years**	
						**Any net**	**ITN**
**ITN Programmes**							
**Eritrea**	2.9	2001–05	900,000	2,000,000	3.7	Nav	59
**Togo**	5.3	2004	900,000	0	6.5	Nav	54
**Malawi**	12.2	1999–05	4,700,000	500,000	15.7	38	36
**Senegal**	10.0	2000–05	750,000	250,000	6.2	14	7
**Tanzania**	35.7	2002–05	6,400,000	7,800,000	30.5	41	28
**IRS Programmes**							

**KwaZulu-Natal**	0.6	1997–99	300,000 structures		2.2	High	High
**Mozambique**	0.8	1999–01	150,000 structures		1.0	> 95% structures	> 95% structures

Nav = not available. ITN = insecticide-treated net.

The ITN programmes also adopted different strategies for the provision of re-treatments for existing nets: Eritrea – free re-treatment through community level campaigns, Malawi – commercial sector cost recovery sales in urban areas, Senegal – partially subsidized private retail sector promotion, and Tanzania – cost recovery private retail sector sale and free delivery to pregnant women at antenatal care visits. Unfortunately, empirical cost evidence was only available for a single country where long-lasting insecticidal nets (LLINs) were introduced, doing away with the need for re-treatment (Togo).

The two IRS programmes were chosen because they were large African programmes and cost data were available for both. They represented: (1) a programme funded locally (KwaZulu-Natal, South Africa) and (2) an international intervention funded by donors and a public-private partnership (Lubombo Spatial Development Initiative – LSDI, Southern Mozambique).

The full programme descriptions are available elsewhere [25].

### Costs

The data on costs were either collected retrospectively for the purpose of the study from financial and operational records (Eritrea, Tanzania, Senegal, Malawi), or taken from raw data sets or published studies which could be adapted to this framework (KwaZulu-Natal, Mozambique, Malawi, Togo). The collection of cost data covered different periods between 1996–2005 (Table 1).

Where possible, the ingredients approach was used: inputs were identified, valued, and classified into activity categories. Where this approach was not possible, either because the information was deemed too sensitive (typically for salaries) or was not available in adequate detail, aggregated expenditures were used. All costs were converted to United States Dollars (USD) based on official yearly average exchange rates for the period during which the costs were incurred (excepting Togo where the exchange rate for the month when most expenditure occurred was used). All costs were adjusted for inflation to 2005 prices using the US gross domestic product deflator [26]. Some costs were estimated using the WHO Choosing Interventions which are Cost-Effective (WHO-CHOICE) unit cost and activity database, specifically those for public sector inputs in Tanzania [27]. No adjustments for purchasing power have been made, and in some cases this may result in problems of comparability across countries due to differences in country specific price levels.

Where possible, both financial and economic costs were collected in order to both estimate the financing requirements for programmes and to examine their efficiency. Financial costs represent purely monetary flows, while economic costs represent the value (or opportunity cost) of all resources necessary to implement a given intervention. Only economic costs are presented here as they are considered the appropriate tool for comparisons of programme efficiency. Financial costs are available elsewhere [25].

A modified provider perspective was used; travel or time costs to users, or other household-level costs or cost savings have not been included. However, the direct costs of net purchases incurred by users have been included where the nets were partially subsidized or sold at full cost. Double counting was avoided by excluding the provider costs which were offset by these user fees. Details on included costs are presented in Table 2.

**Table 2:** Types of costs included in the analysis of the ITN and IRS programmes.

	**ITN Programmes**	**IRS Programmes**
**Capital cost**	Buildings	Buildings
	Vehicles	Vehicles
	ITNs (retail cost & subsidies)^1^	Sprayers
	Other equipment	Other equipment
	Start-up costs^2^	
**Recurrent cost**	Insecticide (when separable from nets)	Insecticide
	Personnel	Personnel
	Fuel/maintenance	Fuel/maintenance
	Management cost/training & meetings	Management cost/training & meetings
	Office/warehouse rental	Office/warehouse rental
	Supplies/overheads	Supplies/overheads
	Recurrent building costs	Recurrent building costs
	Advertising and promotion^3^	Basic evaluation and monitoring (excluding specific research costs)
	Basic evaluation and monitoring (excluding specific research costs)	

^1^Procurement costs in Eritrea and Togo, subsidies plus user payments in Senegal, Tanzania, and Malawi.

^2^Economic costs which arose before outputs of those programmes occurred; these costs apply in Tanzania, Senegal and Malawi; they accounted for fewer than 3% of total economic costs in all cases.

^3^Information, education and communication, health promotion, direct or indirect advertising and promotion subsidies or other similar activities.

### Costing scenarios

The base case costing scenarios relied on the following set of assumptions: a discount rate of 3% was applied to capital costs; nets were assumed to last for three years (physical lifetime), but the effect of initial treatment as well as of subsequent re-treatments were assumed to provide only one year of protection (protective lifetime) [28]; fifty percent of the nets delivered were assumed to be used by children under five years of age and only one child was assumed to sleep under each of these nets on a given night. These are believed to be conservative assumptions [19,29]. The cost of nets was based on the cost, insurance and freight (*c.i.f*.) price of the nets or on the full retail price paid by users plus any subsidies (direct or via vouchers) as estimated by survey data (Senegal and Tanzania) or key informant interviews and project records (Senegal and Malawi). For IRS, it was assumed that the given number of annual spraying rounds (one or two) protected an entire household for one year, with perfect post-spraying compliance (no re-plastering of walls). Reported coverage by IRS was always very high, over 95% at the time of data collection in Mozambique, though high levels of re-plastering have been reported in some IRS interventions (Table 1) [30,31].

Several alternative scenarios were also calculated. One involved the delivery of conventional ITNs in Togo, where only long-lasting insecticidal nets (LLINs) were actually distributed. The other programmes distributed a majority of conventional nets and therefore the costs and outputs in Togo were recalculated assuming a net cost of only USD 3.00 instead of the USD 4.33 paid per LLIN [16,32]. Other alternative scenarios estimated the potential impact of LLIN use on the cost-effectiveness of the programmes. These scenarios were estimated in two ways. The first approach was simply to change the net parameters, including physical lifetime (three or five years), protective lifetime (three or five years) and cost (USD 5.00 to USD 7.00) to values believed to be representative of available LLINs, while not changing the properties, costs or benefits associated with re-treatments [31-34]. The second approach used the same changes for the nets but removed the benefits and commodity costs associated with re-treatments. While it might be reasonable to expect that re-treatments will not be delivered in a LLIN programme, this dual approach was required due to difficulty in separating management and other costs associated with re-treatment. Thus, comparing the two approaches helped us to identify biases in the comparison between programmes due to different re-treatment approaches as well as to better quantify the potential benefits from a shift to LLINs, which would probably result in the discontinuation of most re-treatment activities. One way sensitivity analysis was conducted on all cost estimates.

### Outputs

Two main output measures were used for ITN programmes: (1) number of nets delivered, and (2) number of re-treatments performed. These measures were used to calculate a third combined output measure: treated net years of protection (TNY), assuming that either a re-treatment or a new conventional ITN provided one potential year of protection for anyone sleeping under the net. For IRS programmes two related outputs have been measured: (1) number of persons of any age protected, and (2) number of under-five children protected. Both calculations were made by applying the reported coverage rates to the total population of the sprayed areas – based on census information adjusted for population growth [35,36].

### Outcomes

In order to combine cost data with public health impact, two outcomes were considered. First, the impact of ITNs and IRS on child mortality was estimated. Country-specific estimates were not available but robust impact estimates are available for ITNs from a Cochrane review [37]: ITN use in a high endemicity and high coverage situation averts 5.5 child deaths per 1,000 child-years. For IRS, however, unbiased impact estimates are scarce and have not been systematically reviewed. This is a recognized problem and a Cochrane review is currently investigating this issue [38]. In the absence of a better data set for IRS, and because the impact of ITNs and IRS was found to be similar in the five available randomized comparisons [1,39], the same estimate of impact for both ITNs and IRS (i.e. 5.5 child deaths averted per 1,000 child-years of use) was applied.

Secondly, disability-adjusted life years (DALYs) averted, discounted at 3%, were calculated based on years of life lost due to malaria-specific child mortality. These calculations exclude: (1) DALYs due to disability, and (2) DALYs lost in persons over five years of age. In highly endemic areas the burden of malaria is largely dominated by mortality in children under five years [40]. In addition, quantitative data on the effects of the two interventions are very limited for older children and adults [40,41]. In areas of low malaria transmission or epidemic-prone areas, the burden of disease is more evenly spread over the different age groups and these assumptions do not hold. All child deaths were treated as infant deaths and assigned a value of 33 DALYs lost for each death [42]. This is also a conservative choice as estimates based on deaths distributed across children from one and four years of age would have yielded a higher number of DALYs averted.

### Cost-effectiveness calculations

In a final analysis, the cost per TNY (for ITNs) and per child protected (for IRS) were combined with the impact estimates to produce comparable cost-effectiveness ratios for both interventions. For calculation of impact estimates for ITNs, the number of treated net years (TNYs) delivered was adjusted for net wastage and usage among children by assuming that only 50% of delivered nets (or re-treatments) would be used by under five children and that only one child would sleep under each of these nets. These assumptions are examined in the sensitivity analysis.

The full set of country-specific operational and costing results is available in an unpublished report [25]; selected results only are presented here.

## Results

Tanzania and Malawi were the largest programmes and also the most expensive ones, with a total economic cost of 30.5 and 15.7 million dollars for the periods under review (Table 1). All the ITN programmes were larger in terms of population protected than the two IRS programmes. When the scale of the largest annual ITN delivery is compared to the size of the population at risk for the particular country [43], Togo and Malawi had the highest ITN to population ratios (1:6) and (1:8), respectively, while Senegal had the lowest (1:30). According to the most recent data available for each country, Eritrea had the highest reported ITN usage rate among children under five years of age, while Senegal had the lowest. Both IRS programmes reported very high coverage rates in the targeted areas.

### Economic costs for conventional ITNs

Annualized economic costs per conventional ITN distributed varied from USD 3.23 in Togo (or USD 2.75 if the net price is set to USD 3.00 instead of USD 4.33 – see methods) to USD 8.05 in Senegal (Table 3). Costs per treated net year (TNY) were lower in some cases due to the inclusion of re-treatment of existing nets, which offered additional years of full protection with low commodity costs (approximately USD 0.30. Both measures (cost per ITN and per TNY) include the cost of re-treatments. Hence, the costs per TNY delivered by conventional ITN programmes ranged from USD 1.21 in Eritrea to USD 6.05 in Senegal. It is important to note that higher costs in Senegal were driven to a large extent by higher net costs in the retail sector.

**Table 3:** Average annual economic costs for ITN and IRS programmes.

**ITN programmes**	**Average annual cost per ITN distributed**	**Average annual cost per TNY**	Cost per death averted**	Cost per DALY averted**
Eritrea	3.98	1.21	438/1,449	13/44
Togo	3.23	3.23	1,174	36
Togo*	2.75	2.75	998	30
Malawi	3.36	3.04	1,105/1,222	33/37
Senegal	8.05	6.05	2,199/2,926	67/89
Tanzania	4.80	2.17	788/1,745	24/53

**IRS programmes**	**Cost per person protected (whole population)**	**Cost per under-five child protected**	**Cost per death averted**	**Cost per DALY averted**

KwaZulu-Natal	3.27	23.96	4,357	132
Mozambique	3.90	21.63	3,933	119

*Assuming an average net cost of USD 3 instead of USD 4.33 (the actual cost incurred for LLINs by the campaign).

**In paired numbers the left value includes protection from re-treatment kits

There were important differences in the composition of costs for each programme. The percentage of the total cost born by the programme providers ranged widely: Togo 100%, Eritrea 83%, Malawi 69%, Tanzania and Senegal 45%. When a pure provider perspective is taken by removing all costs paid directly by users of nets, the annualized costs to the provider per net distributed were fairly similar between sites (range USD 2.75–3.63; for detailed results see [25].

Additional calculations for the year during which the programme delivered the largest number of nets were not substantially different from the cost data presented in Table 3[25]. However, the programmes varied greatly in scale (200,000 to approximately three million ITNs per year) and this is likely to contribute to the observed differences in unit costs.

### Economic costs for IRS

In both IRS programmes cost data were only available for one or two years of operations, after the start of the programmes. As a result, it was not possible to measure start-up costs. The annualized costs per person protected for IRS were USD 3.27 in KwaZulu Natal and USD 3.90 in Mozambique (Table 3). However, when only children under five years were included in the denominator for IRS (see methods section) the cost per under five child protected by IRS was substantially higher: USD 23.96 per child protected in KwaZulu Natal and USD 21.63 in Mozambique.

### Sensitivity analysis: one way results

Sensitivity analysis was carried out on the main assumptions and parameters used to calculate cost to output and cost-effectiveness ratios. Discount rate, physical lifetime of nets and costs of insecticide or re-treatment kits all had relatively small effects on the results, however, other parameters were more important, and fit into two groups: (1) those relating to net properties (cost or protective lifetime) and (2) those related to net usage. For programmes involving retail net sales the attribution of commercial sales to programme activities also played an important role. All results were in the expected direction [25].

For the IRS programmes the discount rate also had relatively little effect on the results of the analysis, as did a change in the population growth rate. Several parameters had larger effects on the results, especially the compliance of the population with spraying. Additionally, the cost of adding or removing one of the annual spray rounds or of switching types of insecticides had large effects on the cost-effectiveness of the programmes. Longer transmission seasons or shorter-lived insecticides would require additional spray rounds. The same applies if, for political or resistance reasons, a shorter-acting insecticide were used; more spray rounds imply a higher cost (and potentially higher refusal rates).

### Sensitivity analysis: LLIN cost scenarios

Table 4 shows the annualized economic cost per unit delivered for long-lasting insecticidal nets (LLINs) with three years duration and a cost of USD 5.00. In most settings, annualized costs per net distributed were higher than for conventional nets because of the higher initial purchase price. Senegal was the exception because the commodity costs observed in the programme were much higher than in other programmes. In Togo, the costs changed little because the programme only delivered such LLINs. The annualized delivery costs ranged from USD 3.47 in Togo to USD 7.75 in Eritrea. The cost per TNY was lower than for conventional nets in most cases, ranging from USD 1.46 in Eritrea to USD 2.64 in Senegal (when re-treatments were dropped: USD 2.04 in Malawi to USD 4.14 in Senegal).

**Table 4:** Average annual economic costs for ITN (3-year LLIN) programmes.

**ITN programme**	Average annual cost per LLIN distributed*	Average annual cost per TNY*	Cost per death averted*	Cost per DALY averted*
Eritrea	7.75/7.28	1.46/2.43	531/882	16/27
Togo	3.47	2.37	862	26
Malawi	5.18/4.50	2.19/2.04	798/743	24/23
Senegal	7.58	2.64/4.14	960/1,503	29/46
Tanzania	6.04/5.36	1.83/2.39	664/870	20/26

* In paired numbers the left value includes protection from re-treatment kits while the right value results from removing both protection and commodity costs related to re-treatment kits (see main text for details).

Assumes all nets are conventional ITNs. 2005 USD. TNY = treated net-year. DALY = disability-adjusted life year.

The annualized cost per delivered 5-year LLIN costing USD 7.00 generally fell compared to the three-year scenario, though the differences were not large (Table 5). They ranged from USD 3.23 in Togo to USD 10.08 in Eritrea (when re-treatments are dropped: USD 3.23 in Togo to USD 9.60 in Eritrea). The cost per TNY was also lower than for conventional nets or for three-year LLINs, ranging from USD 1.38 in Eritrea to USD 1.90 in Togo (when re-treatments are dropped: USD 1.69 in Malawi to USD 3.25 in Senegal).

**Table 5:** Average annual economic costs for ITN (5-year LLIN) programmes.

**ITN programme**	Average annual cost per LLIN distributed*	Average annual cost per TNY*	Cost per death averted*	Cost per DALY averted*
Eritrea	10.08/9.60	1.38/1.92	502/698	15/21
Togo	3.23	1.90	692	21
Malawi	5.05/4.36	1.79/1.69	651/616	20/19
Senegal	7.36/6.96	1.67/3.25	606/1,181	18/36
Tanzania	5.74/5.06	1.62/2.28	588/828	18/25

* In paired numbers the left value includes protection from re-treatment kits while the right value results from removing both protection and commodity costs related to re-treatment kits (see main text for details).

Assumes all nets are long-lasting insecticidal nets (LLIN) with **5 years **duration and a cost of USD 7. 2005 USD. TNY = treated net-year. DALY = disability-adjusted life year.

### Cost-effectiveness of ITNs, LLINs and IRS for child mortality prevention

The results for targeted conventional nets are shown in Table 3, the cost per death averted ranged from USD 438 to USD 2,199. The cost per DALY averted was below USD 100 in all cases. Under scenarios with targeted LLINs with three-year physical and protective lifetimes (Table 4), the range of cost per death and DALY averted was lower than in conventional ITN scenarios, although when re-treatment benefits and commodity costs were excluded Tanzania and Eritrea did not show cost-effectiveness improvements. For targeted LLINs with five-year physical and protective lifetimes (Table 5), the cost per death averted in all cases showed cost-effectiveness improvements when compared to the three-year LLIN scenarios.

For IRS, the cost per death averted ranged from USD 3,933 in Mozambique to USD 4,357 in KwaZulu-Natal (Table 3). The cost per DALY averted ranged from USD 119 to USD 132.

**Figure 1:**
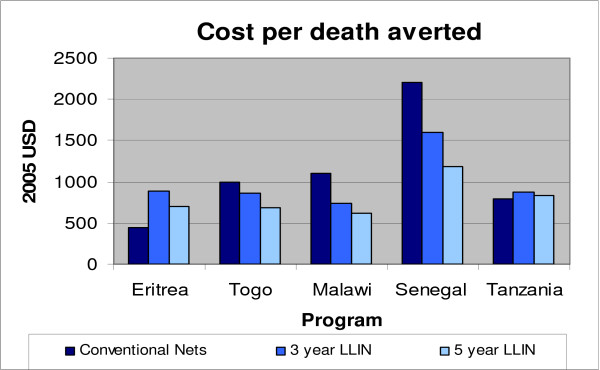
**Cost-effectiveness results of shifting to long-lasting insecticidal nets**. Cost-effectiveness results of shifting to three-year (USD 5) or five-year (USD 7) long-lasting insecticidal nets (LLINs) relative to conventional ITN estimates. LLIN CE estimates do not include protection from re-treatment kits but may include some costs associated with their distribution (see main text).

LLINs, when targeted to high-risk groups in highly endemic areas appear more cost-effective than conventional nets and also more cost-effective than IRS delivered population-wide. Interestingly, the only circumstances in which LLINs did not clearly improve the cost-effectiveness of treated net programmes were in Eritrea and Tanzania (Figure 1). In Eritrea the initial commodity costs for nets were substantially lower than in other settings and in both countries the numbers of re-treatments delivered compared to nets delivered was high.

### Sensitivity analysis: usage of nets by children

A major difference between ITNs and IRS is that the latter intervention cannot be targeted to high-risk demographic groups (pregnant women and small children), since IRS has to be applied to a large proportion of all houses in a geographic area to be effective. This has implications for total cost and also cost-effectiveness, which was explored in a sensitivity analysis. In the base analysis, only one child per net was allowed and it was assumed that only 50% of nets were used to protect children. In sensitivity analysis this usage parameter had an important effect on the cost-effectiveness of every ITN programme because it altered the number of child years of protection provided without changing the costs. At lower usage levels (below 20–30%), the cost-effectiveness of net programmes (especially with conventional nets) resembled that of IRS programmes [25].

An important element that is not considered here for lack of reliable empirical evidence is how coverage of the intervention relates to its health impact. So far, few large-scale vector control programmes have been evaluated reliably with regard to health impact. However, it is well described that with ITN coverage above 50% there is a substantial community effect [44,45], while impact is reduced at lower coverage levels. Since all programmes aim at a high coverage rate (60–80%) and since all reliable impact data has been gathered under this level of coverage [37], it seemed best to consider only one set of impact values.

## Discussion

### Main findings

These results confirm previous work showing that vector control for malaria in sub-Saharan Africa is extremely cost-effective, as even with conservative assumptions the cost per death averted was typically below USD 1,000 for LLINs. Clearly, the move from conventional ITNs to LLINs needs to be effected as soon as possible. In this review, all major types of programmes were covered, with the exception of programmes distributing free LLINs to the whole population (and not only special risk groups such as children) – as promoted recently by WHO [28]. No such programmes had taken place at the time of this work and, therefore, no data could be collected on this strategy. Although the implications of such a large change in the scale of ITN activities are difficult to quantify at present, it should be possible to estimate the cost of such an approach on the basis of this data.

In all programmes under review the cost per treated net-year was surprisingly close in the LLIN scenarios, indicating that programme managers do have real options. However, cost-effectiveness is only one criterion for comparing strategies and decision makers must also consider other important aspects including: (1) the value of continuous promotion (such as found in social marketing programmes) versus a more intermittent approach; (2) the potential of a strategy to strengthen clinics/health facilities and improve uptake of antenatal care or immunization services; (3) the total cost of the strategy in relation to available resources; (4) the equity implications of each strategy; (5) which stakeholders and sectors bear the burden of a given strategy; (6) the opportunity cost in relation to other available health interventions.

It appears that the most cost-effective means of preventing child deaths from malaria is through successfully targeting LLINs to under-five children, while still achieving a relatively high coverage in the rest of the population. If nets were to only protect children little community effect would be realized as children represent less than 20% of the population in sub-Saharan Africa [45]. Under any scenario these findings suggest that LLINs are more cost-effective in high endemicity settings compared to IRS. In areas of year round transmission, especially those with limited physical and human infrastructure, LLINs are also likely to be more feasible than IRS. By contrast, IRS is competitive with ITNs and may be the better option in areas where few spray rounds are required due to either short transmission seasons, the use of inexpensive but long lasting insecticides (such as DDT), or in epidemic prone areas and other situations where good geographic and temporal targeting is possible [46].

Economies of scale and scope need to be considered, as they may have significant effects on the unit cost per ITN by creating efficiencies within the supply chain or by improving demand through the integration of ITN delivery or promotion with a wider range of desirable products or services. In this study, the effects were found to be complex and not clearly present in all reviewed programmes. In Malawi this effect is well documented and the cost per unit in the 5^th ^year of operation fell to approximately 30% of the first year [14]. To some extent, differences in scope and scale may also confound these comparisons given the large differences in the operational scale of each programme as well as relative to the context in which they operate.

### Limitations in the comparability of vector control programmes

In making inter-country comparisons, uncontrollable differences in infrastructure, society, culture, and other variables can induce bias. This study presents selected case studies believed to be representative of different ITN and IRS programmes and delivery approaches in sub-Saharan Africa. However, there may be significant variations in the performance and costs of implementation of these strategies in other settings. Also, in comparing cost information other limitations inevitably arise because of the nature and timing of large scale implementation. For example, it was not possible to compare data sets corresponding to the exact same time period, either temporally or in the history of the programme, nor was it possible to completely control for differences in the scale of programmes. Similarly, it was not possible to control perfectly for variations in price level, although all five ITN countries fit the World Bank definition of a low-income country (GNI per capita below USD 905 in 2006). New country-specific impact data at varying levels of coverage and over the long-term could improve the impact predictions, although ultimately all malaria vector control programmes will aim towards a high coverage of LLINs and hence these differences might not ultimately be marked. Additionally, only the economic costs of programmes are presented here and though this can give guidance in and towards the selection of strategies, decisions must be made in each setting, in the light of both ecological and epidemiological factors as well as local health systems and resources.

### Health system effects

Prevention of malaria can lead to a drastic reduction in the number of patients in health facilities, reducing pressure on over-stretched health facilities and thus benefit the whole health system. The provision of free or low-cost nets at health facilities (through vouchers or as a direct donation) may also be an enticement to pregnant women and mothers to use preventive services. In Malawi a portion of income received from the sale of highly subsidized nets is retained at the facility level and may act as an incentive for staff. On the other hand, comprehensive vector control programmes can burden weak health systems with new activities and lead to additional problems.

ITN programmes with their range of strategic options and possible interactions with non-health sectors appeared more flexible in their demands on the health system than the IRS programmes examined – though some recent IRS programmes, in Bioko Island, Uganda, and Zanzibar have shown that this burden may be shifted to NGOs or commercial organizations [47]. In any case, vector control scale-up requires a capacity expansion in the preventive health care delivery sector. All of the ITN case studies examined here depended to some extent on public sector involvement (though levels of public input were highly variable). Table 6 attempts to summarize qualitatively the demands of the seven vector control programmes on three sectors (public, commercial, non-governmental) based on the results of interviews, document reviews and costing. In the case of the two programmes with a high commercial sector involvement (Tanzania and Senegal), reliance on this sector comes with profit incentives for the actors involved.

**Table 6:** Level of involvement of public, private and non-governmental (NGO) sectors in vector control programmes.

**Programme**	**Public sector**	**Commercial sector**	**NGO sector**
Eritrea	◆◆◆		◆
Togo	◆◆		◆◆◆
Malawi	◆◆	◆◆	◆◆
Senegal	◆	◆◆◆	◆◆
Tanzania	◆◆	◆◆◆	◆◆
KwaZulu-Natal	◆◆◆		
Mozambique	◆◆◆	◆	◆

IRS programmes require a high level of expertise in entomology and management, which might put overwhelming demands to public systems in the face of the generally low availability of trained personnel in many endemic settings. However, a programme can also help to develop local capacity in these areas. The South African example has demonstrated the many ways in which vector control programmes can contribute not only to the reduction of disease but also to the development of local capacity and the training of technicians and scientists.

### Coverage and timing

Major differences were seen in both the levels of coverage achieved in the various ITN programmes, as well as in the duration of time that was required to achieve these coverage levels, or alternatively the time over which they were able to maintain coverage levels. The coverage levels achieved generally correlated with the price charged to users for ITNs: those programmes which delivered nets to users freely achieving the highest measured coverage levels and those with user charges reporting lower coverage and usage. The time to achieve increases in coverage was also generally much faster in programmes without user charges, but only one programme, Eritrea's, allowed for the examination of the long term implications of maintaining coverage with no user charges. While coverage in Eritrea has been successfully maintained for several years without user charges the overall cost per ITN was higher than in several other programmes. The free ITN programmes appeared to be highly effective at achieving higher coverage more quickly than those with user charges, while maintaining lower or competitive cost-effectiveness.

### "Catch-up" versus "keep-up"

Currently, there is a consensus for ITNs that both "catch-up" (to rapidly increase ITN coverage) and "keep-up" (to maintain high ITN coverage) strategies are required in each country [2,48]. Integrated vaccination/ITN campaigns such as those carried out in Togo (studied here), Mozambique, Niger and other settings have achieved good coverage rates (50–60%) within a short period of time [21,49]. Such campaigns can therefore be seen as serving the initial need for "catch-up" in ITN usage levels. However, there is also a strong need for "keep-up" programmes to maintain high net usage levels, especially in newly pregnant women and newborns. Three of the ITN programmes reviewed tended to represent "keep-up" strategies (Malawi, Senegal, Tanzania) while one (Eritrea) mixed both. Recent work in Ghana [49,50] and on the Kenyan ITN programme [51] have highlighted the complementarity of both "catch up" and "keep-up" approaches in achieving high ITN coverage and impressive health impact. Further information and effort will be required to determine which methods can be the most effective and cost-effective to "keep-up" coverage over time.

### Financing

None of the ITN programmes appear to be independently financially sustainable. Even the largely commercial systems, such as the SMARTNET programme in Tanzania or the NetMark programme in Senegal, had substantial donor input and would be unlikely to continue operating at the same scale without continuing donor funding. However, all the programmes under review appeared to be operationally feasible and sustainable in the presence of continued funding. Additionally, in terms of financing, particular attention should be paid to pure provider costs which are more reflective of the inputs of programmes, donors or health ministries because they exclude costs which are borne specifically by users. These costs were similar across the ITN programmes examined here [25].

## Conclusion

These findings confirm that large-scale delivery of ITNs and IRS in sub-Saharan Africa is feasible and highly cost-effective using a range of strategies. Delivery of LLINs through campaigns provides a highly cost-effective and achievable method for rapidly improving ITN coverage. However, many other options exist for ITN programming, some well suited to maintain coverage levels after campaigns. IRS, or a combination of ITNs and IRS, remain attractive and viable options in some settings. Given that sustainable high-level funding appears to be available in the long-term through new global financing mechanisms, every malaria endemic country should aim to upscale their vector control programmes as rapidly and sustainably as possible.

## Funding

The United States Agency for International Development (USAID), the Research Triangle Institute, and the Bill and Melinda Gates Foundation provided funding for this research. These organizations played no role in the design or conduct of the study nor did they play any role in the analysis or interpretation of the results.

## Competing interests

N. Brown is employed by the Swiss Tropical Institute in Tanzania and was involved in the implementation of the Tanzanian National Voucher Scheme. D. Chavasse and J. Justino are employed by Population Services International and were involved in the implementation of the Malawi programme, D. Chavasse was also involved in the implementation of the SMARTNET programme in Tanzania. R. Maharaj is employed by the Medical Research Council of South Africa and is involved in the South African IRS programme as well as in the Lubombo Spatial Development Initiative. T. Ghebremeskel and M. Zerom are employed by the National Malaria Control Programme of Eritrea and are involved in the implementation of the Eritrean ITN programme. D. McGuire, J.M. Urrutia and F. Sakho are employed by the Academy for Educational Development and were involved in the implementation of the NetMark Senegal programme.

## Authors' contributions

JY, CL, KH and FT took the lead in the design, planning and analysis of the inter-country comparisons, as well as in the writing of the manuscript. NB and J-AM were involved in the design, data collection, analysis and writing up of the Tanzania case study; DC, WS and JJ were involved in the design, data collection, analysis and writing up of the Malawi case study; LC and RM were involved in the design, data collection, analysis and writing up of the Mozambique case study; ME, DHM and VW were involved in the design, data collection, analysis and writing up of the Togo case study; TG and MZ were involved in the design, data collection, analysis and writing up of the Eritrea case study; CG and RM were involved in the design, data collection, analysis and writing up of the South Africa case study; JMU and FS and DMcG were involved in the design, data collection, analysis and writing up of the Senegal case study. All authors contributed to the writing of the manuscript and reviewed the final version before submission.

Brian Sharp passed away in April 2007. He was a key driver behind the planning and execution of this work and we would like to honour his memory by listing him as an author.

## Role of funder

The funder of this study played no role in the design, analysis or write-up of the study.
